# Estimating total human deaths from rabies: A statistical framework leveraging reports of rabies transmission from deceased organ and tissue donors

**DOI:** 10.1371/journal.pntd.0014731

**Published:** 2026-09-21

**Authors:** Younjung Kim, Junjie Chen, Alex Yan, Tarek Alrefae, Sumali Bajaj, Christl A. Donnelly

**Affiliations:** 1 Department of Statistics, University of Oxford, Oxford, United Kingdom; 2 Pandemic Sciences Institute, University of Oxford, Oxford, United Kingdom; Colorado State University, UNITED STATES OF AMERICA

## Abstract

Estimating the global burden of human rabies is notoriously challenging because the infection is often misdiagnosed or not reported. Although rare, instances of donor-derived human-to-human rabies transmission have been identified after organ or tissue transplantation. Here, we present a statistical framework leveraging these instances to estimate both the probability that a death from rabies is diagnosed for an infected individual and the number of total deaths from rabies. Of the 33 canine-rabies-endemic and 13 canine-rabies-free countries assessed, rabies was identified among deceased donors after transplantation in China (n = 4) between 2015 and 2024 and the United States of America (US) (n = 3) between 2000 and 2024. Assuming the same proportion of deaths from rabies among deceased donors and decedents in the general population, we estimated that China and the US diagnosed rabies in only 28.7% (95%CI: 14.8–56.5%) and 6.1% (95%CI: 2.4–21.1%) of rabies decedents, respectively, during the study years. In 2024, these corresponded to 581 (95%CI: 308–1,056) and 75 (95%CI: 19–242) estimated total deaths from rabies compared to 167 and 4 reported deaths from rabies, respectively. Although parameters were imprecisely estimated for the remaining countries, for countries with a relatively large number of deceased donors, our framework provided informative upper confidence bounds on total deaths from rabies, which can inform the design of surveillance and control measures. For example, in 2023, this upper bound was estimated to be 2,227 deaths from rabies for India. Parameter estimates were sensitive to the assumed relative risk of rabies between deceased donors and decedents in the general population, highlighting the need for future research to estimate this quantity and improve inference within our framework. Our findings show substantial underreporting of human deaths from rabies in both canine-rabies-endemic and canine-rabies-free countries, underscoring the need to strengthen surveillance and diagnostic capacity and raise risk awareness.

## Author summary

Rabies is a fatal zoonotic viral disease responsible for a substantial mortality burden in humans globally. However, estimating the true global burden of rabies infection in humans is notoriously challenging. In this study, we estimated the total number of deaths from rabies and the probability of diagnosing a death from rabies for an infected individual in canine-rabies-endemic and canine-rabies-free countries. To do so, we used a statistical framework that leverages instances of donor-derived human-to-human rabies transmission events documented in China and the US, alongside data on reported deaths from rabies, deceased donors, and all-cause deaths. We also applied this framework to other countries with no documentation of such events to assess its broader utility and to provide upper bounds on their total number of deaths from rabies. Our results suggest substantial underreporting occurs not only in canine-rabies-endemic countries but also in the US, where most human rabies infections are caused by wildlife exposure, including bats. While the exact estimates of underreporting are sensitive to underlying model assumptions, our study emphasises the importance of enhancing rabies risk awareness and strengthening rabies surveillance and diagnostic capacity.

## Introduction

Rabies is a fatal infectious disease affecting mammals including dogs, cats, livestock, and wildlife, caused by neurotropic viruses in the family *Rhabdoviridae*, genus *Lyssavirus* [1]. Without pre- or post-exposure prophylaxis, rabies causes near 100% fatality in humans [1]. A substantial number of human deaths are reported globally, predominantly through bites, scratches, or mucosal exposure by dogs [2]. However, human rabies is believed to be substantially underreported. First, symptoms are often misdiagnosed as other neurological conditions. Second, infected individuals often do not recall or report their contacts with animals. Third, the disease disproportionately affects marginalised communities with limited access to healthcare for post-exposure prophylaxis and diagnostic testing, particularly in regions where canine rabies is endemic [3]. Such underreporting complicates robust estimation of the human rabies burden and thus the allocation of surveillance and control resources to reduce it.

Among existing approaches to estimate the burden of human rabies, models that are based on a probability tree are most commonly used, with a particular focus on dog-mediated transmission [3–6]. Based on data from 2000 to 2014, Hampson et al (2015) [3] reported the estimate of 59,000 dog-mediated human deaths from rabies per annum, and this point estimate is widely cited to highlight the substantial global public health impact by canine rabies [2]. A key strength of these models is that they explicitly represent the sequence of conditional events leading to human rabies infection, including the incidence of dog bites, the probability that a bite is from a rabid dog, the probability that an exposed individual does not receive a post-exposure prophylaxis, and the probability that the individual develops rabies. However, data to inform these parameters are difficult to obtain, particularly because most human deaths from rabies occur in resource-limited settings [3]. As a result, model fitting often relies on assumptions informed by data from other regions or time periods. The issue is that these alternative data may not be readily available or externally valid and can therefore introduce bias into model estimates. Hampson et al (2015) [3] explicitly acknowledged this limitation through their sensitivity analyses.

Other approaches to estimate the burden of human rabies also focus on dog-mediated transmission and include interviewing the family or close associates of individuals who died from an unknown cause and conducting surveys of animal bites or suspected human rabies cases, often using snowball sampling [7–11]. However, rabies is frequently misdiagnosed as other neurological diseases (while the reverse cannot be excluded in settings with limited diagnostic capacity for neurological diseases). Also, causative animal bites are commonly not recognised or reported because the incubation period is long and variable, typically 2–3 months, but ranging from weeks to one year [2]. As a result, while these approaches provide opportunities to understand the local context of rabies transmission, they have limitations for burden estimation.

In this study, we propose a straightforward complementary statistical framework that estimates the burden of human deaths from rabies from a different perspective. This work is motivated by multiple reports of rabies infection in deceased donors that were not recognised at the time of death and transplantation but were confirmed retrospectively after rabies was diagnosed in organ or tissue recipients [12]. Deceased donors represent only a small fraction of all deaths and rabies causes near 100% mortality without pre- or post-exposure prophylaxis. These characteristics suggest that the confirmation of initially undiagnosed deaths from rabies through donor-derived transmission provides a distinctive statistical signal that can help infer the underlying burden of human rabies mortality in these settings. Most of these events have been documented in China [13–16] and the United States (US) [17–20], which have markedly different rabies epidemiology. Canine rabies is endemic in China [3], whereas the US is free of canine rabies and human cases are largely associated with transmission from wildlife reservoirs, such as bats, raccoons, skunks, and foxes [21].

Our framework leverages data on (a) the number of deaths from rabies identified among deceased donors whose rabies infection was only confirmed after transplantation and onward transmission, together with (b) the number of reported deaths from rabies, as well as (c) the number of deceased donors and (d) the number of all-cause deaths. We apply our framework to all countries for which the required data are available from publicly available global datasets, including both canine-rabies-endemic and canine-rabies-free countries. By fitting a unified model to all of these data, we jointly estimate the underlying burden of human rabies and the rabies diagnostic performance for each country.

For countries lacking documented donor-derived rabies transmission events, we still include their results primarily for analytical completeness. Furthermore, depending on the statistical signal from the remaining data, the framework can still yield an informative upper confidence bound on total human deaths from rabies. We explicitly discuss the factors to consider when interpreting the results from these countries.

## Methods

### Data

The data were collated as follows.

First, a literature search for donor-derived rabies transmission events was conducted on PubMed using the query “Rabies AND (Donor* OR Recipient* OR Transplant*)”. An equivalent search was performed on the China National Knowledge Infrastructure (CNKI) using the corresponding Chinese terms: “狂犬病 AND (移植 OR 受者 OR 受体 OR 捐献者 OR 供者 OR 供体)”.

Second, annual numbers of reported human deaths from rabies were obtained from the World Health Organization (WHO) Global Health Observatory (GHO) data repository (accessed December 22, 2025) [22]. The data were available for the period 2010–2024; however, for the US, we supplemented these with additional data for 2000–2009 [21,23], as human rabies cases and the data described below were reported systematically during this period in the country.

Third, for countries included in the human rabies mortality dataset, annual numbers of deceased donors were sourced from the Global Observatory on Donation and Transplantation (GODT) (accessed January 6, 2026) [24] and International Registry in Organ Donation and Transplantation (IRODaT) (accessed January 18, 2026) [25], using IRODaT values when data were not available in GODT.

Finally, annual numbers of total deaths were obtained from the United Nations Population Division Data Portal (accessed January 6, 2026) [26].

All data were merged into a single dataset. Only countries with data on human deaths from rabies and deceased donors available for at least one year during the study period were included. Countries were classified as canine-rabies-free or canine-rabies-endemic, based on the US Centers for Disease Control and Prevention (CDC) assessment (accessed January 19, 2026) [27]. Canine-rabies-free countries were still included in the analysis if they had at least one human death from rabies, as the parameters estimated by our approach (the probability of rabies diagnosis among infected individuals, and the probability that a death is due to rabies) are as relevant for imported cases as for locally acquired cases.

### Modelling approach

**Fig 1:**
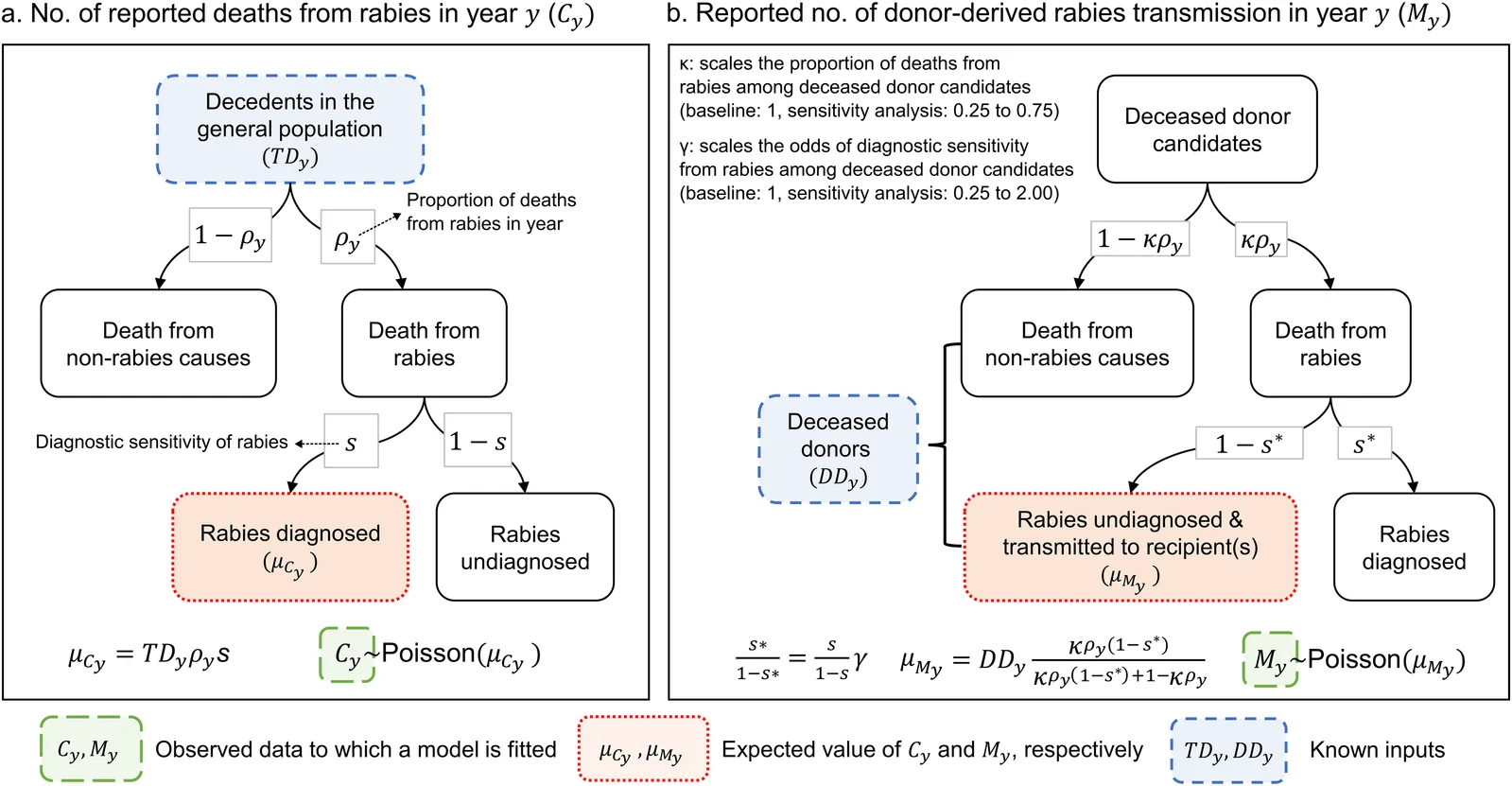
Schematic description of the modelling framework.

Our modelling framework is depicted in Fig 1. The baseline assumptions are: (i) the proportion of deaths from rabies was the same in deceased donor candidates and decedents in the general population, (ii) transplantation from a rabies-infected donor led to rabies infection in at least one recipient and subsequent confirmation of rabies infection in the donor, the probability of diagnosing a death from rabies for an infected individual (diagnostic sensitivity) was (iii) country-specific, (iv) remained the same during the study years, and (v) was the same among deceased donor candidates and decedents in the general population. We performed sensitivity analyses for baseline assumptions i, iii, and v (see Sensitivity analysis).

For each country and for each year y, we modelled the annual number of reported deaths from rabies in the general population,$C_{y}$, and the annual number of deaths from rabies among deceased donors whose rabies infection was unrecognised at the time of death but confirmed after transplantation, $M_{y}$, as independent Poisson counts conditional on the model parameters:


$$\begin{array}{c} C_{y}\sim \text{Poisson}\left(\mu_{Cy}\right) \end{array}$$ Eq. 1

$$\begin{array}{c} M_{y}\sim \text{Poisson}\left(\mu_{My}\right) \end{array}$$ Eq. 2

Here, $\mu_{Cy}$ and $\mu_{My}$ represent the expected value of $C_{y}$ and the expected value of $M_{y}$, respectively.

The Poisson means were specified as:


$$\begin{array}{c} \mu_{Cy}={TD}_{y}\rho_{y}s \end{array}$$ Eq. 3

$$\begin{array}{c} \mu_{My}={DD}_{y}\frac{\rho_{y}(\text{1}-s)}{\rho_{y}(\text{1}-s)+\text{1}-\rho_{y}}={DD}_{y}\frac{\rho_{y}(\text{1}-s)}{\text{1}-\rho_{y}s} \end{array}$$ Eq. 4

Here, s and $\rho_{y}$ were model parameters which were estimated. s represents the probability of diagnosing a death from rabies for an infected individual (“diagnostic sensitivity”), which was assumed to be time-invariant, and $\rho_{y}$ represents the proportion of deaths from rabies among all-cause deaths in year y (“rabies-death proportion”). ${TD}_{y}$ denotes the number of all-cause deaths in year y, and ${DD}_{y}$ the number of deceased donors in year y. ${TD}_{y}$ and ${DD}_{y}$ were known inputs from the data. In Eq. 4, our framework excluded deceased donor candidates who died from and were diagnosed with rabies (along the pathway in Fig 1 from *Deceased donor candidates*, *Death from rabies*, to *Rabies diagnosed*). Excluding these cases gives the proportion of human deaths from rabies among the final deceased donors, ${DD}_{y}$, leading to donor-derived rabies transmission, as $\frac{\rho_{y}(\text{1}-s)}{\rho_{y}(\text{1}-s)+\text{1}-\rho_{y}}$ simplified to $\frac{\rho_{y}(\text{1}-s)}{\text{1}-\rho_{y}s}$ in Eq. 4 (Fig 1). Since the same values of s and $\rho_{y}$ apply to both the candidates and final donors, hereafter for conciseness, we use the term ‘deceased donors’.

Parameters s and $\rho_{y}$ were estimated using maximum profile likelihoods. First, to estimate the diagnostic sensitivity s we evaluated the profile log-likelihood across values of s on a fine grid over (0, 1), $\text{\textbackslash{}text\{\textbackslash{}specialsymfont 𝓁}{\}}_{p}(s)$ (Eq. 5). For each candidate value of s, the year-specific proportion of deaths from rabies ${\hat{\rho }}_{y}(s)$ was estimated numerically using the *optimise* function in R (v. 4.3.3) [28], and $\text{\textbackslash{}text\{\textbackslash{}specialsymfont 𝓁}{\}}_{p}(s)$ was computed (Eq. 5). The grid value maximising $\text{\textbackslash{}text\{\textbackslash{}specialsymfont 𝓁}{\}}_{p}(s)$ was taken as the maximum likelihood estimate $\hat{s}$, and its 95% confidence interval was obtained using likelihood ratio tests where:


$$\begin{array}{c} \text{\textbackslash{}text\{\textbackslash{}specialsymfont 𝓁}{\}}_{p}(s)=\sum_{y=\text{1}}^{Y}\left\{\text{log}\text{Pois}(C_{y};{TD}_{y}{\hat{\rho }}_{y}(s)s\right)+\text{log}\text{Pois}(M_{y};{DD}_{y}\frac{{\hat{\rho }}_{y}(s)(\text{1}-s)}{\text{1}-{\hat{\rho }}_{y}(s)s})\} \end{array}$$ Eq. 5

Second, we also used a profile likelihood approach to estimate the maximum likelihood year-specific proportion of deaths from rabies ${\hat{\rho }}_{y}$. For a given year i, we treated $\rho_{i}$ as the parameter of interest and s and $\rho_{j^{\prime }}$ ($j^{\prime }\neq i)$ as nuisance parameters. To profile $\rho_{i}$, for each candidate value of $\rho^{\text{0}}$ on a grid, we evaluated the profile log-likelihood:


$$\begin{array}{c} \text{\textbackslash{}text\{\textbackslash{}specialsymfont 𝓁}{\}}_{p}\left(\rho^{\text{0}}\right)=\underset{s\in (\text{0},\text{1})}{\text{max}}\{{\text{\textbackslash{}text\{\textbackslash{}specialsymfont 𝓁}}_{i}(s;\}\rho^{\text{0}})+\sum_{j\neq i}{\text{\textbackslash{}text\{\textbackslash{}specialsymfont 𝓁}}_{j}(s;\}{\hat{\rho }}_{j}(s))\} \end{array}$$ Eq. 6

Here, $\text{\textbackslash{}text\{\textbackslash{}specialsymfont 𝓁}{\}}_{i}(s,\rho^{\text{0}})$ is the log-likelihood contribution from year i evaluated at $\rho_{i}=\rho^{\text{0}}$ and, for other years (j≠i), $\rho_{j}$ were set to their conditional maximum likelihood estimate ${\hat{\rho }}_{j}(s)$ for each s. Thus, $\rho^{\text{0}}$ maximising $\text{\textbackslash{}text\{\textbackslash{}specialsymfont 𝓁}{\}}_{p}\left(\rho^{\text{0}}\right)$ was the maximum likelihood estimate ${\hat{\rho }}_{i}$, and its confidence interval was obtained using likelihood ratio tests.

We estimated the number of undiagnosed deaths from rabies in year y,$U_{y}$, defined as:


$$\begin{array}{c} {\hat{U}}_{y}={TD}_{y}{\hat{\rho }}_{y}(\text{1}-\hat{s}) \end{array}$$ Eq. 7

For each year i, we obtained the maximum likelihood estimate ${\hat{U}}_{i}$ by substituting the maximum likelihood estimates $\hat{s}$ and ${\hat{\rho }}_{i}$ derived above. To quantify uncertainty in ${\hat{U}}_{i}$, we computed the minimum and maximum values of $U_{i}$ over the two-dimensional likelihood region $R_{i}$:


$$\begin{array}{c} R_{i}=\{\left(s,\rho_{i}\right):\text{2}(\text{\textbackslash{}text\{\textbackslash{}specialsymfont 𝓁}{\}}_{p,max}-\text{\textbackslash{}text\{\textbackslash{}specialsymfont 𝓁}\}\left(s,\rho_{i}\right))\leq \chi_{\text{1},\text{1}-\alpha }^{\text{2}}\} \end{array}$$ Eq. 8

Here, $\text{\textbackslash{}text\{\textbackslash{}specialsymfont 𝓁}{\}}_{p,max}$ is $\text{\textbackslash{}text\{\textbackslash{}specialsymfont 𝓁}{\}}_{p}\left(\hat{s}\right)$ (see profile likelihood for s above), and $\text{\textbackslash{}text\{\textbackslash{}specialsymfont 𝓁}\}\left(s,\rho_{i}\right)$ is the log-likelihood at $\left(s,\rho_{i}\right)$ with $\rho_{j}={\hat{\rho }}_{j}(s)$ for j≠i (see profile likelihood for$\rho_{y}$ above).

Finally, the estimated total number of deaths from rabies in year y,$T_{y}$, was obtained as


$$\begin{array}{c} {\hat{T}}_{y}{=\hat{U}}_{y}+C_{y} \end{array}$$ Eq. 9

### Sensitivity analysis

First, in the baseline model, we assumed that the proportion of deaths from rabies was the same among the deceased donor and decedents in general populations. However, this assumption might not always hold. For example, socioeconomic status might be associated both with becoming a deceased donor and with factors influencing rabies mortality (e.g., through differences in dog-bite exposure and access/uptake to post-exposure prophylaxis). Donor selection and screening might also exclude individuals with certain clinical presentations (e.g., encephalitis of unknown cause). Therefore, we assessed the impact of this assumption on model estimates by modifying Equation 4 as:


$$\begin{array}{c} \mu_{My}={DD}_{y}\frac{{\kappa \rho }_{y}(\text{1}-s)}{\text{1}-\kappa \rho_{y}s} \end{array}$$ Eq. 10

with varying values of κ (i.e., 0.25, 0.50, 0.75, and 1.00). For example, κ=0.25 indicates that deceased donors have one-quarter the proportion of deaths from rabies compared with decedents in the general population.

Second, our baseline model also assumed that diagnostic sensitivity was identical among deceased donor and decedents in general populations. This assumption might also not apply, depending on the distribution of causes of deaths and resulting heterogeneities in diagnostic protocols taken, or the existence of additional diagnostic requirements for deceased donor eligibility. Unlike our sensitivity analysis on the proportion of deaths from rabies, we could not find empirical information regarding the potential direction or extent of such influence. Therefore, we performed a sensitivity analysis where diagnostic sensitivity s in deceased donors was scaled with varying values of γ in both directions (i.e., 0.25, 0.50, 1.50, and 2.00) relative to the decedents in the general population using the odds. Scaling the odds ensured that s remained within a valid probability range.


$$\begin{array}{c} \frac{s*}{\text{1}-s*}=\frac{s}{\text{1}-s}\gamma \end{array}$$ Eq. 11

$$\begin{array}{c} \mu_{My}={DD}_{y}\frac{\rho_{y}(\text{1}-s^{*})}{\text{1}-\rho_{y}s^{*}} \end{array}$$ Eq. 12

Finally, we noted that deaths from rabies among deceased donors were reported only in China among rabies-endemic countries and only in the US among canine-rabies-free countries. These deaths, in addition to their relatively large numbers of deceased donors, enabled the most precise parameter estimation among the countries investigated. We therefore explored how the proportions of deaths from rabies and consequently the total deaths from rabies estimates would change if the studied countries had the same diagnostic sensitivity (s) as China (for canine-rabies-endemic countries) or the US (for canine-rabies-free countries). To do so, rather than estimating s separately for each country, we fitted the model jointly to data from the countries with the same canine-rabies status (canine-rabies-endemic countries including China or canine-rabies-free countries including the US) assuming a common s across the countries, while still assuming country- and year-specific $\rho_{y}$, using profile likelihoods as described above.

### Model fitting and evaluation

The model was fitted in R (v. 4.3.3) [28] using custom-built code (see Code A and Code B in S1 Files). We assessed model adequacy using a likelihood-ratio goodness-of-fit test against a saturated Poisson model, based on the deviance statistic:


$$\begin{array}{c} D=\text{2}\{\text{\textbackslash{}text\{\textbackslash{}specialsymfont 𝓁}{\}}_{sat}-\text{\textbackslash{}text\{\textbackslash{}specialsymfont 𝓁}{\}}_{p}\left(\hat{\theta }\right)\} \end{array}$$ Eq.13

Here, $\text{\textbackslash{}text\{\textbackslash{}specialsymfont 𝓁}{\}}_{p,sat}$ is the log-likelihood under the saturated model with the Poisson means ($\mu_{Cy}$ and $\mu_{My}$) set to the observed counts ($C_{y}$ and $M_{y}$), and $\text{\textbackslash{}text\{\textbackslash{}specialsymfont 𝓁}{\}}_{p}\left(\hat{\theta }\right)$ is the maximum log-likelihood of the fitted model. With degrees of freedom $df=\text{2}n_{y}-(n_{y}+\text{1})$, where $n_{y}$ is the number of years with data points, a p-value was obtained from the $\chi_{df}^{\text{2}}$ distribution.

Separately, to help assess whether the available data for each country provided sufficient statistical signal to yield informative parameter estimates, we mapped the log-likelihood deviance across the joint parameter space of diagnostic sensitivity (s) and the proportion of deaths from rabies (ρ). For this exploration, ρ was assumed to be constant across the years.

## Results

### Descriptive analysis of the underlying data

The dataset included 46 countries, of which 33 (71.7%) were classified as canine-rabies-endemic. The included countries were in Asia/Eastern Mediterranean (n = 16), the Americas (n = 15), and Europe (n = 13). Only two countries (Algeria and South Africa) were included from Africa in the analysis.

**Fig 2:**
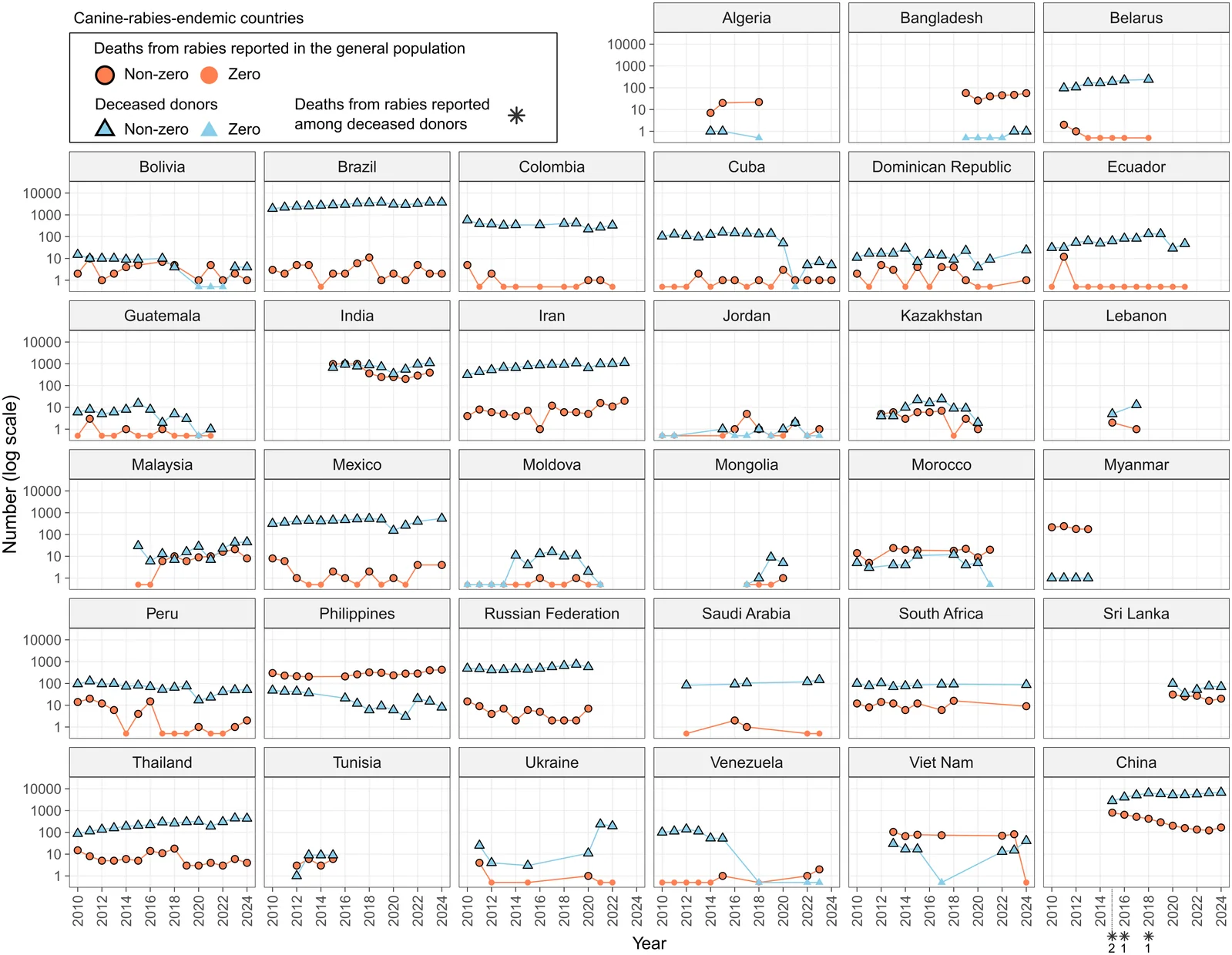
Reported deaths from rabies and deceased donors in canine-rabies-endemic countries. The countries are shown in alphabetical order except for China where donor-derived rabies transmission events were documented. For each country and year, the figure shows (a) the number of deaths from rabies reported in the general population (orange circles) and (b) the number of deceased donors (blue triangles). Only years with data available for both (a) and **(b)**, and therefore analysed, are shown. The y-axis shows (a) and (b) on a log scale; zero values are plotted as points with no border. The x-axis shows years; for China, asterisks mark years with at least one death from rabies among deceased donors whose rabies infection was confirmed after transplantation, with the corresponding counts shown below the asterisks.

**Fig 3:**
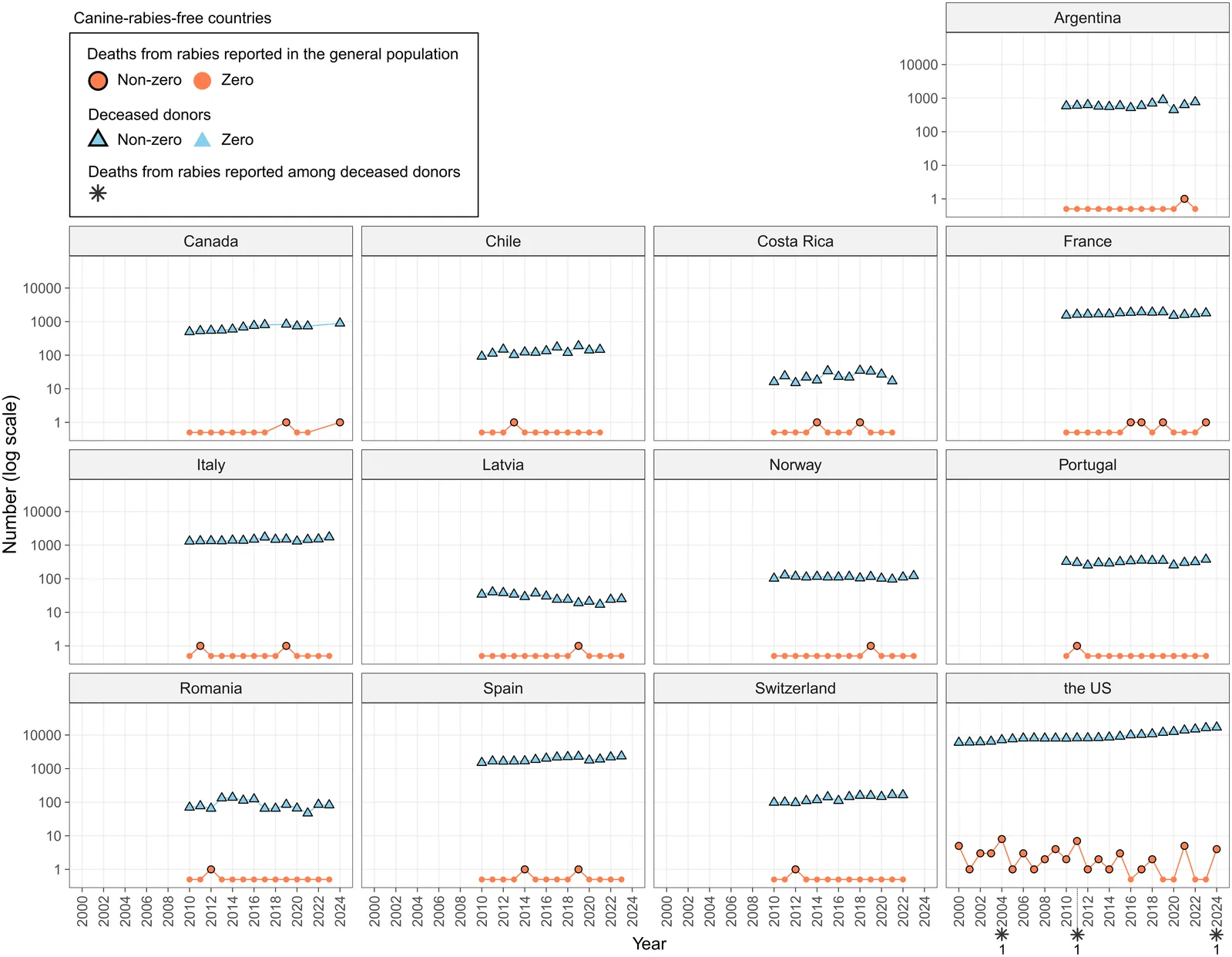
Reported deaths from rabies and deceased donors in canine-rabies-free countries. The countries are shown in alphabetical order except for the US where donor-derived rabies transmission events were documented. For each country and year, the figure shows (a) the number of deaths from rabies reported in the general population (orange circles) and (b) the number of deceased donors (blue triangles). Only years with data available for both (a) and **(b)**, and therefore analysed, are shown. The y-axis shows (a) and (b) on a log scale; zero values are plotted as points with no border. The x-axis shows years; for the US, asterisks mark years with at least one death from rabies among deceased donors whose rabies infection was confirmed after transplantation, with the corresponding counts shown below the asterisks.

The data used for model fitting are shown in Fig 2 for canine-rabies-endemic countries and Fig 3 for canine-rabies-free countries. They include (a) deaths from rabies reported in the general population, (b) deceased donors, and (c) deaths from rabies among deceased donors whose rabies infection was confirmed after transplantation (see S1–S2 Fig for the corresponding values per million all-cause deaths).

The number of reported deaths from rabies varied substantially across canine-rabies-endemic countries. In absolute numbers, India, China, and the Philippines reported markedly higher reported deaths from rabies than other countries (Fig 2), with the Philippines having experienced the highest reported deaths from rabies reported per million all-cause deaths (S1 Fig). In India and China, reported deaths from rabies peaked in the earlier study years and then declined overall, with the decline more pronounced in China until the most recent years (Fig 2). In contrast, no clear declining trend was observed in the Philippines, along with the other canine-rabies-endemic countries. Among canine-rabies-free countries, the US reported deaths from rabies in most years assessed since 2000, with up to eight deaths per year (Fig 3). In the remaining canine-rabies-free countries, deaths from rabies were not reported in most years, with at most one per year when reported (Fig 3).

Deceased donor numbers also varied considerably across canine-rabies-endemic countries (Fig 2). While donor data were available for only a limited number of years for some countries, the data were generally more complete for countries with a large number of reported deaths from rabies, including India, China, and the Philippines (Fig 2). Some canine-rabies-endemic countries reported relatively small numbers of deceased donors compared to reported deaths from rabies. For example, in the Philippines, although deceased donor numbers were consistently reported in most years, they were substantially smaller than reported deaths from rabies in all study years (270 deceased donors versus 3,670 reported deaths from rabies, compared to 6,902 deceased donors versus 4,713 reported deaths from rabies for India, Fig 2). Among canine-rabies-free countries, the deceased donor data were generally more complete, with a clear increasing trend in the US (Fig 3).

Finally, deaths from rabies among deceased donors confirmed after transplantation were reported only in China (four events in 2015, 2016, and 2018) and the US (three events in 2004, 2011, and 2024) during the study years.

### Estimates of human rabies burden and diagnostic sensitivity

Here, we first describe how parameter estimates varied with data availability and model assumptions, and then present results for China, the US, India, and the Philippines as examples, given their contrasting data features and rabies endemicity. Results for other countries are provided in S1 Files.

The point estimates and confidence intervals of the model parameters varied widely across countries (see Figure Collection in S1 Files for country-specific parameter space exploration). This variation was driven primarily by whether deaths from rabies had been reported among deceased donors, as these cases provided an independent statistical signal to distinguish the parameters. The numbers of deaths from rabies and deceased donors also played distinct roles in shaping parameter estimates. First, the number of reported deaths from rabies determined the precision of the estimated proportion of deaths from rabies. Countries with a large number of reported deaths from rabies exhibited tightly defined parameter combinations that were consistent with the data, whereas countries with few reported deaths from rabies displayed wider bands of uncertainty. In contrast, the number of deceased donors determined the upper bound on the estimated proportion of deaths from rabies. In countries with a large number of deceased donors, the data effectively capped the upper bound, providing informative, constrained estimates of the proportion of deaths from rabies. However, in countries with few deceased donors, the confidence region was very wide.

China and the US were the only countries with documented donor-derived transmission events, as well as large deceased donor pools and sufficient reported deaths from rabies, thereby yielding the most well-identified parameter estimates; the US exhibited a wider confidence interval relative to its point estimate than China, reflecting its fewer reported deaths from rabies (see Figure Collection in S1 Files for country-specific parameter space exploration). In contrast, for countries with no reported donor-derived rabies transmission, estimating country-specific diagnostic sensitivity led to point estimates for total deaths from rabies equal to the reported deaths from rabies, and the informativeness of the upper bounds on the estimated proportion of deaths from rabies (and total deaths from rabies) to inform rabies surveillance depended on the numbers of deceased donors and reported deaths from rabies (see Figure Collection in S1 Files for country-specific parameter space exploration).

Parameter estimates also varied systematically with assumptions about (i) how the proportion of deaths from rabies among deceased donors compared with that among decedents in the general population and (ii) whether diagnostic sensitivity for deaths from rabies among those infected was estimated separately for each country or as a shared parameter across countries with the same canine-rabies status. When deceased donors were assumed to have a lower proportion of deaths from rabies than decedents in the general population, the estimated proportion of deaths from rabies among decedents in the general population (and therefore the estimated total deaths from rabies) increased; for China and the US, this shift affected both the point estimates and confidence intervals, whereas for countries with no death from rabies among deceased donors, only the upper bounds increased while the point estimates remained equal to the reported number of deaths from rabies. On the other hand, estimating diagnostic sensitivity as a shared parameter improved parameter identifiability for countries where no deaths from rabies were reported among deceased donors by increasing the proportion of deaths from rabies and reducing its uncertainty. In contrast, for China and the US, with such reports, this approach increased estimated diagnostic sensitivity while reducing the estimated proportions of deaths from rabies and therefore the estimated total deaths from rabies.

**Fig 4:**
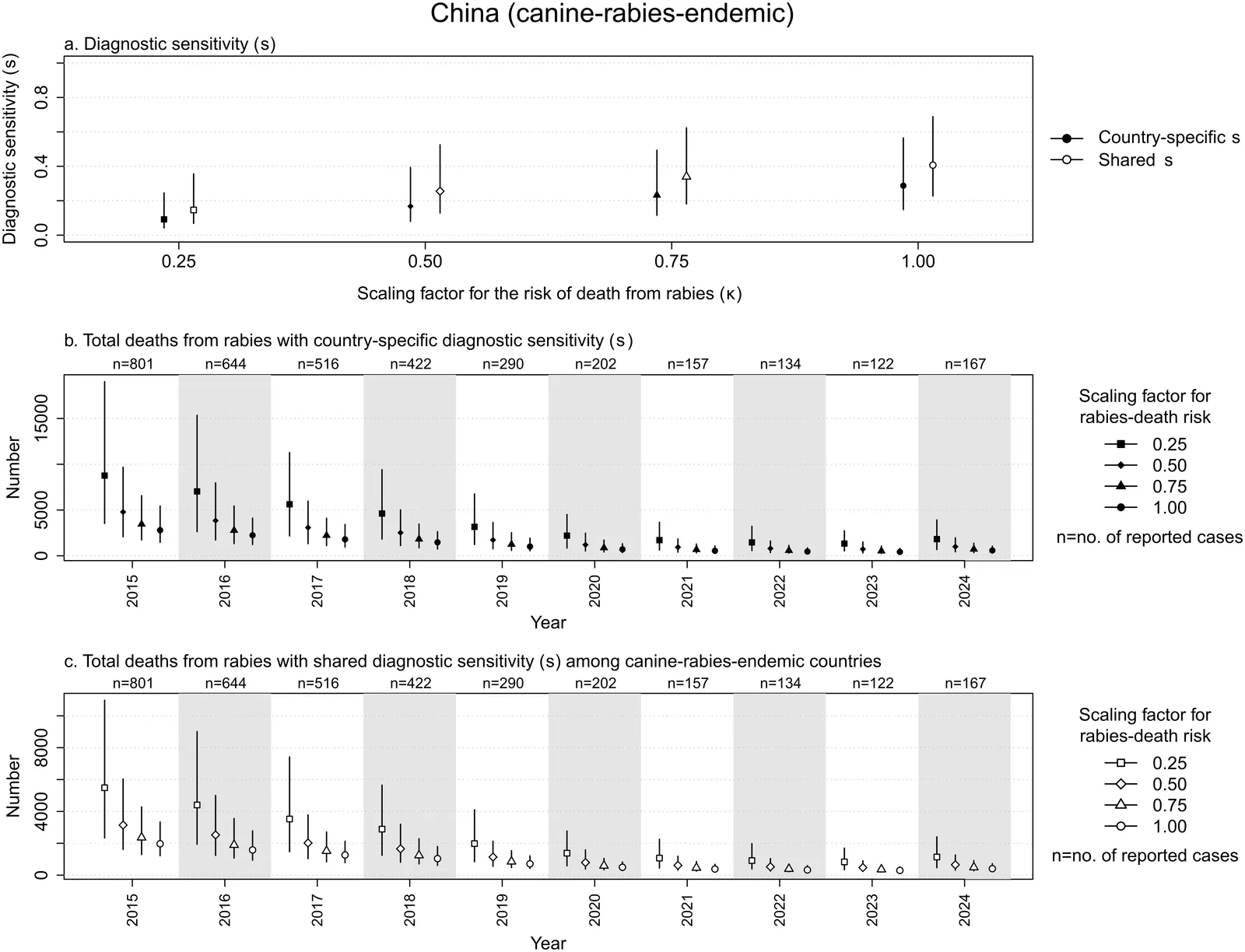
Model estimates for China under alternative assumptions about the proportion of deaths from rabies among deceased donors relative to decedents in the general population (scaling factor κ) and diagnostic sensitivity (s). Panel a shows the estimated diagnostic sensitivity (s) across values of κ, with s estimated either as a country-specific parameter (filled points) or as a shared parameter across canine-rabies-endemic countries (hollow points). Panel b shows the estimated total deaths from rabies using country-specific s, and Panel c shows the estimated total deaths from rabies using s shared across canine-rabies-endemic countries. In all panels, point shapes denote κ values, and vertical lines indicate 95% confidence intervals.

For China, under the baseline assumptions, estimated country-specific diagnostic sensitivity was 28.7% (95%CI: 14.8 to 56.5%; black circle in Fig 4A). When diagnostic sensitivity was modelled as a shared parameter across canine-rabies-endemic countries, it increased to 40.7% (95%CI: 22.8 to 68.8%; white circle in Fig 4A). Assuming a lower proportion of deaths from rabies among deceased donors reduced the estimated country-specific diagnostic sensitivity; when deceased donors were assumed to have one quarter the proportion of deaths from rabies among decedents in the general population, estimated diagnostic sensitivity decreased to 9.2% (95%CI: 4.2 to 24.5%; black square in Fig 4A).

Estimated total deaths from rabies in China showed a declining trend over the study years. Under the baseline assumptions, the estimated total deaths from rabies decreased from 2,792 in 2015 (95%CI: 1,469–5,437) to 581 in 2024 (95%CI: 308–1,056) (black circles in Fig 4B). Assuming a lower proportion of deaths from rabies among deceased donors increased the estimated total deaths from rabies (Fig 4B); with the proportion of deaths from rabies among deceased donors assumed to be one quarter that among decedents in the general population, the estimated total deaths from rabies rose to 1,820 in 2024 (95%CI: 680–3,907). In contrast, when diagnostic sensitivity was estimated as a shared parameter, the estimated total deaths from rabies decreased only slightly (Fig 4C); assuming the same proportion of deaths from rabies for deceased donors and decedents in the general population, the estimated total deaths from rabies decreased from 581 (95%CI: 308–1,056) to 410 (95%CI: 243–726) in 2024.

**Fig 5:**
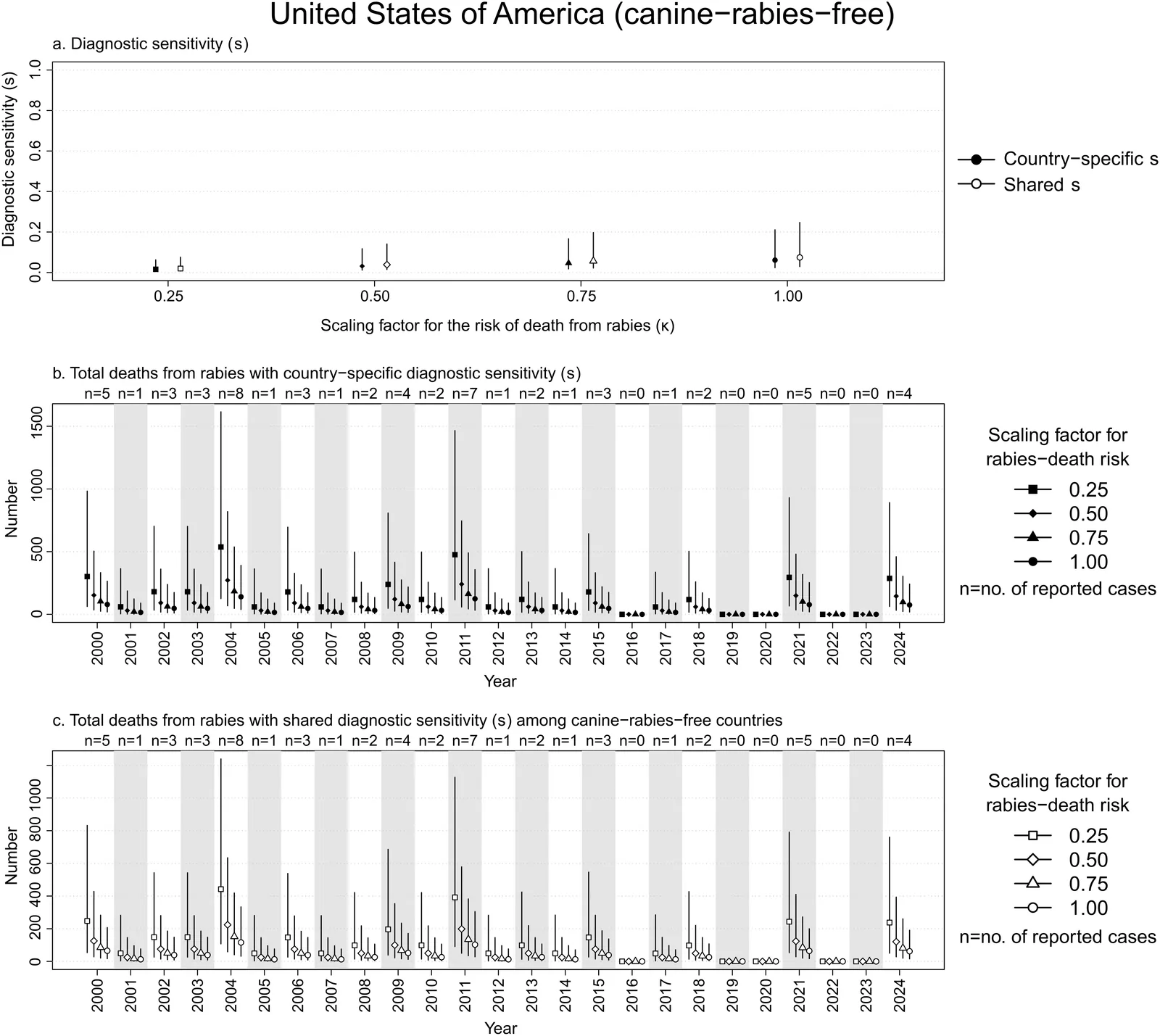
Model estimates for the US under alternative assumptions about the proportion of deaths from rabies among deceased donors relative to decedents in the general population (scaling factor κ) and diagnostic sensitivity (s). Panel a shows the estimated diagnostic sensitivity (s) across values of κ, with s estimated either as a country-specific parameter (filled points) or as a shared parameter across canine-rabies-free countries (hollow points). Panel b shows the estimated total deaths from rabies using country-specific s, and Panel c shows the estimated total deaths from rabies using s shared across canine-rabies-free countries. In all panels, point shapes denote κ values, and vertical lines indicate 95% confidence intervals.

For the US, under the baseline assumptions, the estimated country-specific diagnostic sensitivity was 6.1% (95%CI: 2.4 to 21.1%; black circle in Fig 5A), substantially lower than that for China. When diagnostic sensitivity was estimated as a shared parameter across canine-rabies-free countries, it increased only slightly to 7.4% (95%CI: 2.9 to 24.7%; white circle in Fig 5A). Assuming a lower proportion of deaths from rabies among deceased donors further reduced the estimated country-specific diagnostic sensitivity; when deceased donors were assumed to have one quarter the proportion of deaths from rabies among decedents in the general population, estimated diagnostic sensitivity decreased to 1.6% (95%CI: 0.6 to 6.3%; black square in Fig 5A).

From 2000 to 2024, the total deaths from rabies in the US remained broadly stable, with the point estimates ranging from 0 in years with no reported deaths from rabies to 140 (95%CI: 38–391) in 2004 (black circles in Fig 5B). Assuming a lower proportion of deaths from rabies among deceased donors increased the total deaths from rabies; in 2024, the estimate rose from 75 (95%CI: 19–242) to 287 (95%CI: 63–891) when deceased donors were assumed to have one quarter the proportion among decedents in the general population. When diagnostic sensitivity was estimated as a shared parameter, the estimated total deaths from rabies decreased only slightly (Fig 5C); in 2024, assuming the same proportion of deaths from rabies for deceased donors and decedents in the general population, the total deaths from rabies decreased to 62 (95%CI: 16–191).

**Fig 6:**
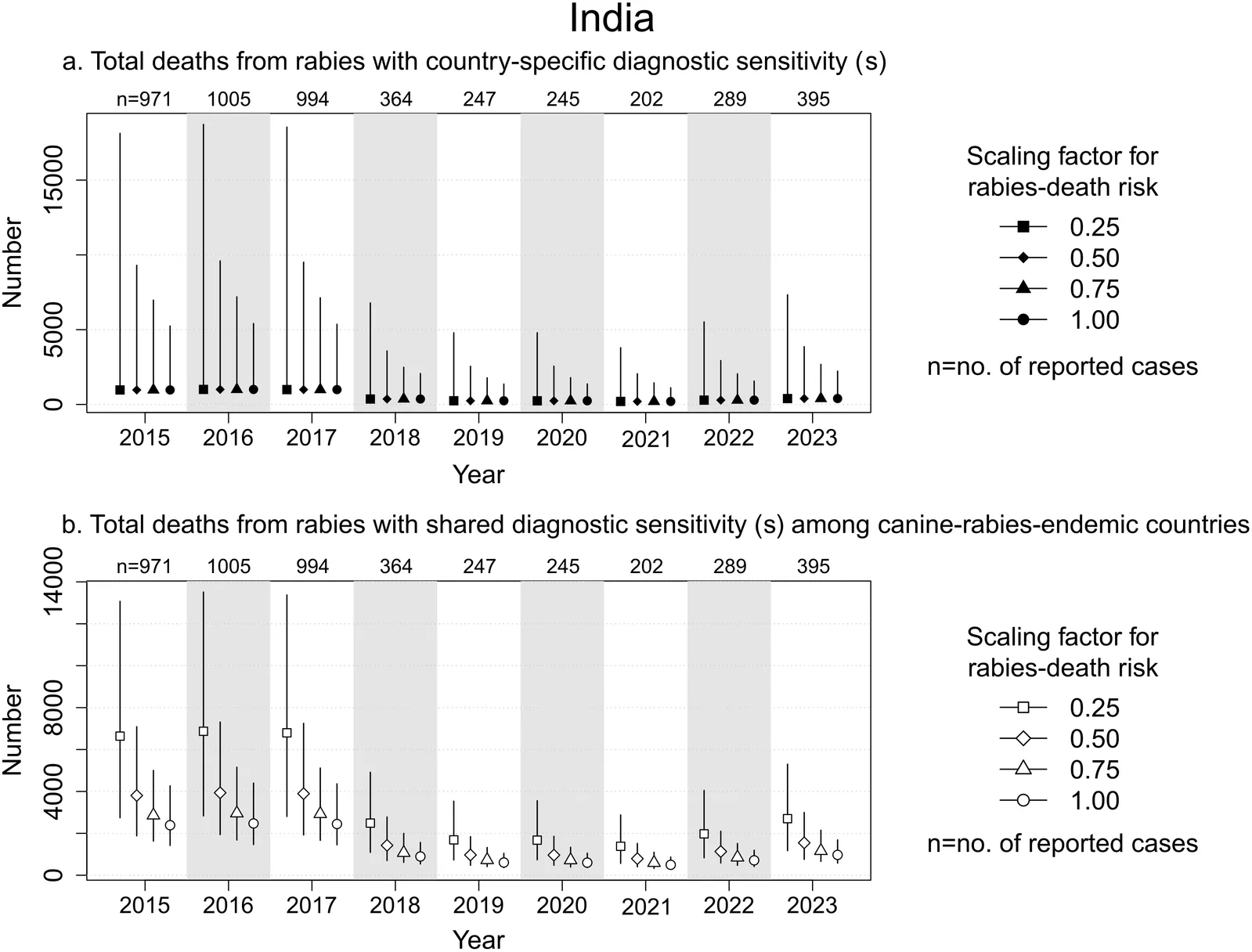
Estimates of total deaths from rabies for India. Panel a shows the estimated total deaths from rabies when country-specific diagnostic sensitivity (s) was estimated, and Panel b shows the estimates when diagnostic sensitivity (s) was estimated as a shared parameter across canine-rabies-endemic countries. In all panels, point shapes denote κ values, which scaled the proportion of deaths from rabies among deceased donors relative to decedents in the general population. Vertical lines indicate 95% confidence intervals.

**Fig 7:**
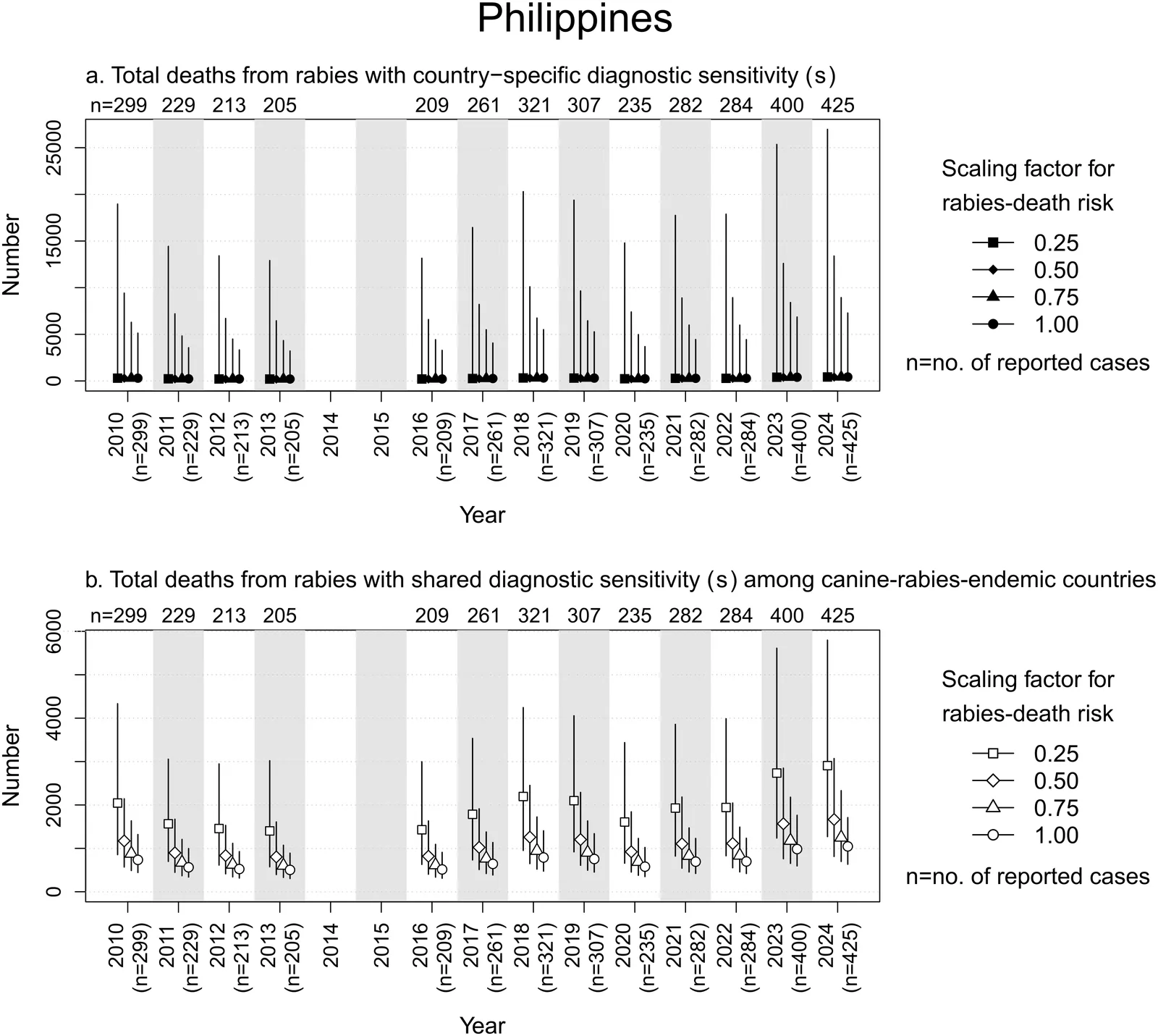
Estimates of total deaths from rabies for the Philippines. Panel a shows the estimated total deaths from rabies when country-specific diagnostic sensitivity (s) was estimated, and Panel b shows the estimates when diagnostic sensitivity (s) was estimated as a shared parameter across canine-rabies-endemic countries. In all panels, point shapes denote κ values, which scaled the risk of death from rabies among deceased donors relative to decedents in the general population. Vertical lines indicate 95% confidence intervals.

For the remaining countries, the absence of deaths from rabies among deceased donors made country-specific diagnostic sensitivity weakly identified, with its point estimates concentrated near the upper bound of 1 and substantial uncertainty (Fig 6 for India and Fig 7 for the Philippines; see country-specific results in S1 Files for other countries). This weak identification propagated to estimates of the proportion of deaths from rabies, pushing their point estimates toward zero while leaving wide confidence intervals. Nonetheless, for countries with a relatively large number deceased donors, the data still provided informative upper bounds on the proportion of deaths from rabies (and thus on total deaths from rabies); if the true proportions were higher than these upper bounds, then, unless diagnostic sensitivity were perfect, we would have observed at least some deaths from rabies among deceased donors confirmed after transplantation.

For India, assuming that the proportion of deaths from rabies was the same in deceased donors and decedents in the general population, the estimated proportions of deaths from rabies decreased significantly from 2015 to 2021 before increasing again through 2023. Under the baseline assumptions, the upper bound on the total deaths from rabies was 5,244 in 2015 and 2,227 in 2023 (Fig 6a). When the model instead assumed one-quarter the proportion of death from rabies among decedents in the general population, the upper bound increased to 18,128 in 2015 and 7,332 in 2023 (Fig 6a). When diagnostic sensitivity was estimated as a shared parameter, point estimates were better identified with reduced uncertainty; the total deaths from rabies were estimated at 971 in 2023 (95%CI: 581–1,687; Fig 6b), with a shared diagnostic sensitivity and assuming the same proportion of deaths from rabies for deceased donors and decedents in the general population.

For the Philippines, the estimated proportions of deaths from rabies were broadly similar over the study years, but increased from 2021 onward, with peak total deaths from rabies estimated in 2024 (Fig 7). Under the baseline assumptions, the upper bound on the total deaths from rabies was 7,282 in 2024. When deceased donors were assumed to have one quarter the proportion of deaths from rabies among decedents in the general population, the upper bound increased to 26,970 in 2024 (Fig 7a). As in India, estimating diagnostic sensitivity as a shared parameter improved identifiability; the total deaths from rabies were estimated at 1,045 in 2024 (95%CI: 634–1,709, Fig 7b).

Finally, for China and the US, scaling up diagnostic sensitivity among deceased donors affected point estimates, but to a substantially lesser extent than varying the proportion of deaths from rabies among deceased donors relative to decedents in the general population. Shifting from a 75% decrease to a 100% increase in the odds of diagnostic sensitivity among deceased donors decreased the estimated diagnostic sensitivity for decedents in the general population from 35.4% (95%CI: 16.5 to 74.7%) to 24.5% (95%CI: 13.3 to 46.9%) in China (S3a Fig), and from 6.4% (95%CI: 2.4 to 24.7%) to 5.8% (95%CI: 2.3 to 18.4%) in the US (S4a Fig), while holding other baseline assumptions constant. In 2024, these shifts corresponded to changes from 471 (95%CI: 223–976) to 681 (95%CI: 355–1,199) in China (S3b Fig), and from 71 (95%CI: 16–233) to 79 (95%CI: 20–232) in the US (S4b Fig). For countries with no reported donor-derived rabies transmission, scaling diagnostic sensitivity among deceased donors had no noticeable impact, except for a few countries (e.g., India and Brazil). In these countries, scaling up the diagnostic sensitivity among deceased donors marginally decreased the lower bound of diagnostic sensitivity among decedents in the general population and increased the upper bound of total deaths from rabies (see country-specific results in S1 Files).

## Discussion

In this study, we present a novel statistical framework that jointly links diagnostic sensitivity for deaths from rabies among infected individuals and the proportion of deaths from rabies to distinct data streams: reported deaths from rabies among decedents in the general population and deaths from rabies identified among deceased donors through donor-derived transmission, alongside the underlying denominators of all-cause deaths and deceased donors.

We show that the framework can estimate both diagnostic sensitivity and the burden of human rabies (parameterised as the proportion of deaths from rabies) with documented donor-derived rabies transmission even when these cases are rare. This, in turn, allows estimation of the number of total deaths from rabies. We demonstrate this for China and the US, which reported four and three deaths from rabies reported among deceased donors, respectively, during the study years.

Our estimates of diagnostic sensitivity and total deaths from rabies for China and the US highlight substantial underdiagnosis, and therefore underreporting, of human deaths from rabies. For China, our results are consistent with prior literature that has estimated the burden of human rabies in canine-rabies-endemic countries using other approaches [3–11]. Given that canine rabies disproportionately affects marginalised communities, particularly in canine-rabies-endemic countries, deaths that occur outside the healthcare system likely drive the estimated low diagnostic sensitivity in China. Supporting this, Qi et al (2018) [29] reported that only 12.2% of rabies cases in Chongqing, a province in China, sought hospital treatment. Additionally, even for patients who present to healthcare providers, misdiagnosis could occur, as most regions affected by canine rabies have limited diagnostic capacity, particularly given that rabies confirmation requires either CSF or brain tissue samples [3].

The low diagnostic sensitivity estimated for the US highlights that underreporting of human rabies infections could also be substantial in countries where infection risk is primarily limited to wildlife exposure, and secondarily to travel to canine-rabies-endemic countries. Our estimates suggest that, relative to the few reported cases, a substantial number of deaths from rabies have been undiagnosed in the US. However, in absolute numbers, these deaths from rabies still represent only a very small fraction of all-cause deaths, which might have contributed to limited rabies risk awareness among both the public and healthcare providers [30,31]. Additionally, most rabies infections in the US are associated with bat exposures [21,32], and bat bites can result in minor or unrecognisable wounds. Also combined with a long incubation period ranging weeks to one year, this might reduce healthcare seeking after exposure and hinder recall of relevant animal contacts [31,33]. While these factors indicate challenges in improving diagnostic sensitivity in the US, they also emphasise that maintaining national laboratory and surveillance infrastructure is imperative, with further efforts to enhance rabies risk awareness and implement predefined criteria to trigger rabies diagnostic testing (e.g., testing for all unexplained encephalitis).

For both China and the US, assuming a lower proportion of deaths from rabies among deceased donors than decedents in the general population further reduced the estimated diagnostic sensitivity, with a more pronounced effect in China. While the risk of rabies infection is likely associated with geographical, occupational, and socioeconomic factors, we do not have data to help quantify the relative risk between the two groups for each country. Thus, assuming an equal risk as the baseline scenario may be seen as appropriate until evidence of differential risks is available. In alternative scenarios, deceased donors might have a lower risk of death from rabies mainly via the following factors. First, rabies disproportionately affects marginalised communities, particularly in canine-rabies-endemic countries. Individuals in these communities may be less likely to become deceased donors, potentially due to limited access to healthcare facilities capable of organ transplantation and lower awareness of organ donation [34,35]. As a result, deceased donors may disproportionately originate from less marginalised communities and thus have a lower risk of death from rabies than decedents in the general population. This scenario may be more plausible in China, where canine rabies remains endemic, than in the US, where most rabies infections in the US occur via bat exposures in home or non-occupational settings, rather than contexts strongly linked to marginalisation [21,32]. Second, donor selection and screening may exclude individuals who die with certain clinical presentations, such as encephalitis of unknown cause, including the overt clinical signs with furious rabies [36]. However, the documentation of donor-derived rabies transmissions in China and the US suggests that implementation of such processes (in the form of protocols, guidelines, or recommendations) may be imperfect in practice, particularly when clinical presentation is non-differential to other neurological diseases (e.g., non-overt clinical manifestation with paralytic rabies) and/or exposure history is incomplete, features that are common in rabies [37].

Among the limited prior estimates for China, Hampson et al (2015) [3] reported a point estimate of 6,002 deaths per year (95%CI: 992–10,742) using 2000–2014 data. In fact, this value lies between our point estimates for 2015 (the year closest to their data period) under assumptions that the proportion of deaths from rabies among deceased donors is one-quarter to one-half of that among decedents in the general population (4,785–8,776 deaths, respectively). If the estimate by Hampson et al (2015) [3] is reasonably accurate, this comparison suggests that the relative proportion of deaths from rabies among deceased donors in China is plausibly between these two assumptions.

For countries without documented donor-derived transmission events but with active organ and tissue transplantation programmes (and therefore a relatively large number of deceased donors), the estimated upper bounds on total deaths from rabies may still help inform the design of surveillance and control measures. In those countries, a high upper confidence bound indicates that the data remain compatible with substantial under-ascertainment, thereby supporting conservative public health approaches, for example, maintaining surveillance and diagnostic testing criteria in high-risk settings. However, in countries where organ and tissue transplantations are not commonly performed (and therefore few deceased donors), interpreting these upper bounds requires careful consideration due to a limited statistical signal for parameter estimation.

India had relatively large numbers of both deceased donors and reported deaths from rabies, suggesting that their upper bounds on the proportion (and therefore the total number) of deaths from rabies) would represent an informative maximum burden of human rabies infection in the country. Compared to estimates from earlier studies, the upper bound reported by Thangaraj et al (2025) [6] was 7,350 deaths per year using 2022–2023 data. This value was comparable to our upper bound estimates for the same years under assumptions that the proportion of deaths from rabies among deceased donors is one-quarter to one-half of that among decedents in the general population (4,215–8,679 for 2022, and 6,102–11,589 for 2023, respectively). The upper bound reported by Hampson et al (2015) [3] using 2000–2014 data was 55,421 deaths per year, substantially higher than our upper-bound estimate of 29,197 for 2015 (the year closest to their data period) under the one-quarter relative-risk assumption for deceased donors. If the underlying burden of rabies remained similar across these years and assuming Hampson et al (2015) [3]’s estimates are reasonably accurate, this comparison would suggest an even lower relative rabies-death risk among deceased donors than investigated here.

Interestingly, for the Philippines, assuming a country-specific diagnostic sensitivity and across the explored range of scaling factors, our upper-bound estimates throughout the study years (2010–2024) remained substantially higher than the upper bound reported by Hampson et al (2015) [3] (1,083 deaths per year, using 2000–2014 data), even when assuming an equal proportion of deaths from rabies among deceased donors and decedents in the general population. Notably, their point estimate of 168 deaths per year was consistently lower than officially reported deaths from rabies in the country, including during 2010–2014 (205–299 deaths per year) although the observed numbers were within the 95% confidence limits (129–1,083 deaths per year). However, interpreting our estimates for the Philippines requires careful consideration as its number of deceased donors, though reported consistently throughout the study period, was substantially lower than its number of reported deaths from rabies.

In the absence of documented donor-derived rabies transmission events, we show that parameter identifiability improves when information on diagnostic sensitivity is available as a shared parameter. One approach to obtain such information would be post-mortem confirmatory diagnostic testing in a representative sample of decedents meeting predefined rabies-like clinical criteria, comparing laboratory-confirmed results with the recorded cause of death. Multiple reports of rabies being initially missed at the times of death in both deceased donors and non-donors suggest that carefully designed surveillance studies could help estimate diagnostic sensitivity and better quantify human rabies burden within our framework. However, such studies may be difficult in low-resource settings, particularly where a non-negligible fraction of deaths from rabies occurs at home.

An alternative approach is to borrow information from other countries with similar healthcare access and diagnostic capacity. Treating diagnostic sensitivity as a shared parameter across countries with the same canine-rabies status, we show that identifiability can improve substantially. Nonetheless, healthcare access and diagnostic capacity may vary substantially among countries with the same canine-rabies status. Moreover, China and the US were the only countries with documented donor-derived rabies transmission events in our data, which limits the utility of this approach to settings with very different healthcare systems.

The interpretation of findings from our framework warrants several additional considerations, which must be considered separately for each country as they are likely heterogenous across different settings. First, our framework assumes that transplantation from a rabies-infected donor resulted in rabies infection among at least one recipient leading to retrospective confirmation of rabies infection in the donor; transplanted organs and tissues would be expected to contain virus because systemic infection is likely to be present at the time of transplantation. Nevertheless, we cannot fully exclude rare scenarios in which only uninfected organ or tissue is transplanted or rabies in recipients is not recognised (and, consequently, the donor infection remains unidentified). If such events occur but are not recorded, their omission from the observed data could lead to an overestimation of diagnostic sensitivity, and consequently underestimation of total deaths from rabies.

Second, we assumed constant diagnostic sensitivity across the study years within each country. In practice, diagnostic sensitivity may change if the epidemiological context shifts, for example, through changes in rabies-risk awareness and diagnostic capacity. Although diagnostic sensitivity was well identified for China and the US in our framework, temporal variation may be plausible for China given the declining trend in deaths from rabies in recent years, whereas it may be less apparent in the US where reported deaths from rabies remained broadly stable over the study years. Proxy indicators that could inform time-varying diagnostic sensitivity include periodic surveys of rabies-risk awareness, trends in the supply and use of post-exposure prophylaxis, and changes in rabies prevention and control policy. Incorporating such information, however, would require additional assumptions to disentangle their association with changes in underlying rabies risks to humans. Furthermore, diagnostic sensitivity could also be different between deceased donors and decedents in the general population. Although our sensitivity analysis showed that its impact is minimal within the explored range of differences, the direction and extent of this variation could vary substantially across settings, thereby warranting context-specific application of our methodology and interpretation of its findings.

Finally, future research should better characterise the relative risk of death from rabies between deceased donors and decedents in the general population to improve inference when the framework is applied. One priority would be to assess differential rabies risk, by comparing animal-contact rates in populations with similar geographical, occupational, and socioeconomic profiles to deceased donors with decedents in the general population. In addition, guidelines for donor selection and their implementation in practice, as well as the rabies surveillance system should be carefully evaluated. One approach would be to compare the distribution of causes of death among deceased donors with that among decedents in the general population, focusing on conditions relevant to rabies (e.g., encephalitis of unknown cause), to assess whether donor selection disproportionately excludes such presentations.

In conclusion, our study presents a statistical framework to estimate diagnostic sensitivity for rabies diagnosis and rabies burden in humans. It shows that reports of deaths from rabies among deceased donors, although few, can provide critical information for parameter estimation, as demonstrated for China and the US. When such reports are absent, the framework can still provide upper bounds on total rabies burden, which may help inform public health policy. Leveraging routinely available public data, this framework offers a straightforward complementary approach, and its estimates could be further improved through studies that better inform the underlying assumptions.

## Supporting information

S1 Fig Deaths from rabies and deceased donors in canine-rabies-endemic countries per million all-cause deaths. The countries are shown in alphabetical order except for China where donor-derived deaths from rabies were confirmed. For each country and year, the figure shows (a) the number of deaths from rabies reported in the general population (orange circles) and (b) the number of deceased donors (blue triangles), both per million all-cause deaths. Only years with data available for both (a) and (b), and therefore analysed, are shown. The y-axis shows (a) and (b) on a log scale; zero values are plotted as points with no border. The x-axis shows years; for China, asterisks mark years with at least one death from rabies among deceased donors whose rabies infection was confirmed after transplantation, with the corresponding counts shown below the asterisks. (DOCX)

S2 Fig Deaths from rabies and deceased donors in canine-rabies-free countries per million all-cause deaths.The countries are shown in alphabetical order except for the US where donor-derived deaths from rabies were confirmed. For each country and year, the figure shows (a) the number of deaths from rabies reported in the general population (orange circles) and (b) the number of deceased donors (blue triangles), both per million all-cause deaths. Only years with data available for both (a) and (b), and therefore analysed, are shown. The y-axis shows (a) and (b) on a log scale; zero values are plotted as points with no border. The x-axis shows years; for the US, asterisks mark years with at least one death from rabies among deceased donors whose rabies infection was confirmed after transplantation, with the corresponding counts are shown below the asterisks. (DOCX)

S3 Fig Model estimates for China under alternative assumptions about the proportion of deaths from rabies among deceased donors relative to decedents in the general population (scaling factor) and diagnostic sensitivity (s). Panel a shows the estimated diagnostic sensitivity (s) across values of γ, with s estimated either as a country-specific parameter (filled points) or as a shared parameter across canine-rabies-endemic countries (hollow points). Panel b shows the estimated total deaths from rabies using country-specific s, and Panel c shows the estimated total deaths from rabies using s shared across canine-rabies-endemic countries. In all panels, point shapes denote values, and vertical lines indicate 95% confidence intervals. (DOCX)

S4 Fig Model estimates for the US under alternative assumptions about the diagnostic sensitivity among deceased donors relative to decedents in the general population (scaling factor) and diagnostic sensitivity (s). Panel a shows the estimated diagnostic sensitivity among decedents in the general population (s) across values of γ, with s estimated either as a country-specific parameter (filled points) or as a shared parameter across canine-rabies-free countries (hollow points). Panel b1-2 shows the estimated total deaths from rabies using country-specific s, and Panel c1-2 shows the estimated total deaths from rabies using s shared across canine-rabies-free countries. In all panels, point shapes denote values, and vertical lines indicate 95% confidence intervals. (DOCX)

S1 Files Supplementary files including model codes (Code_A.R Code_B.R) and additional country specific results (Figure Collection.pdf, README.docx, **Result A folder,** **Result B folder**).
(ZIP)

S1 Checklist Checklist. (PDF)
