# Supplementary material for: Estimating total human deaths from rabies: A statistical framework leveraging reports of rabies transmission from deceased organ and tissue donors
Source: PLoS Negl Trop Dis. 2026 Sep 21;20(9):e0014731. doi: 10.1371/journal.pntd.0014731 (PMC13592719; doi:10.1371/journal.pntd.0014731)
Supplement: Supplementary file 3 [file pntd.0014731.s002.docx]

S2 Fig Deaths from rabies and deceased donors in canine-rabies-free countries per million all-cause deaths. The countries are shown in alphabetical order except for the US where donor-derived deaths from rabies were confirmed. For each country and year, the figure shows (a) the number of deaths from rabies reported in the general population (orange circles) and (b) the number of deceased donors (blue triangles), both per million all-cause deaths. Only years with data available for both (a) and (b), and therefore analysed, are shown. The y-axis shows (a) and (b) on a log scale; zero values are plotted as points with no border. The x-axis shows years; for the US, asterisks mark years with at least one death from rabies among deceased donors whose rabies infection was confirmed after transplantation, with the corresponding counts are shown below the asterisks.
