# Supplementary material for: Estimating total human deaths from rabies: A statistical framework leveraging reports of rabies transmission from deceased organ and tissue donors
Source: PLoS Negl Trop Dis. 2026 Sep 21;20(9):e0014731. doi: 10.1371/journal.pntd.0014731 (PMC13592719; doi:10.1371/journal.pntd.0014731)
Supplement: Supplementary file 4 [file pntd.0014731.s003.docx]

S3 Fig. Model estimates for China under alternative assumptions about the proportion of deaths from rabies among deceased donors relative to decedents in the general population (scaling factor $\gamma$) and diagnostic sensitivity (s). Panel a shows the estimated diagnostic sensitivity (s) across values of $\gamma$, with s estimated either as a country-specific parameter (filled points) or as a shared parameter across canine-rabies-endemic countries (hollow points). Panel b shows the estimated total deaths from rabies using country-specific s, and Panel c shows the estimated total deaths from rabies using s shared across canine-rabies-endemic countries. In all panels, point shapes denote $\gamma$ values, and vertical lines indicate 95% confidence intervals.
